# Liraglutide Therapy in Obese Patients Alters Macrophage Phenotype and Decreases Their Tumor Necrosis Factor Alpha Release and Oxidative Stress Markers—A Pilot Study

**DOI:** 10.3390/metabo14100554

**Published:** 2024-10-16

**Authors:** Łukasz Bułdak, Aleksandra Bołdys, Estera Skudrzyk, Grzegorz Machnik, Bogusław Okopień

**Affiliations:** Department of Internal Medicine and Clinical Pharmacology, Faculty of Medical Sciences, Medical University of Silesia in Katowice, Medyków 18, 40-752 Katowice, Poland

**Keywords:** liraglutide, obesity, macrophage, alternative activation, GLP-1

## Abstract

**Introduction**: Obesity is one of the major healthcare challenges. It affects one in eight people around the world and leads to several comorbidities, including type 2 diabetes, hyperlipidemia, and arterial hypertension. GLP-1 analogs have become major players in the therapy of obesity, leading to significant weight loss in patients. However, benefits resulting from their usage seem to be greater than simple appetite reduction and glucose-lowering potential. Recent data show better cardiovascular outcomes, which are connected with the improvements in the course of atherosclerosis. Macrophages are crucial cells in the forming and progression of atherosclerotic lesions. Previously, it was shown that in vitro treatment with GLP-1 analogs can affect macrophage phenotype, but there is a paucity of in vivo data. **Objective**: To evaluate the influence of in vivo treatment with liraglutide on basic phenotypic and functional markers of macrophages. **Methods**: Basic phenotypic features were assessed (including inducible nitric oxide synthase, arginase 1 and mannose receptors), proinflammatory cytokine (IL-1β, TNFα) release, and oxidative stress markers (reactive oxygen species, malondialdehyde) in macrophages obtained prior and after 3-month therapy with liraglutide in patients with obesity. **Results**: Three-month treatment with subcutaneous liraglutide resulted in the alteration of macrophage phenotype toward alternative activation (M2) with accompanying reduction in the TNFα release and diminished oxidative stress markers. **Conclusions**: Our results show that macrophages in patients treated with GLP-1 can alter their phenotype and function. Those findings may at least partly explain the pleiotropic beneficial cardiovascular effects seen in subjects treated with GLP-1 analogs.

## 1. Introduction

The World Health Organization estimates that one in eight people is obese [1]. Therefore, it must be clearly stated that obesity is becoming a pandemic nowadays. It is a disease that leads to secondary complications (e.g., hyperlipidemia, hypertension, diabetes, atherosclerosis, metabolic-associated steatotic liver disease—MASLD) that reduce patients’ life span and quality of life. Currently, there are several options to treat obesity, including surgical and pharmacological interventions. The most promising seems to be incretin mimetics that improve patients’ efforts to reduce body weight (e.g., liraglutide, semaglutide). During the treatment with glucagon-like peptide type 1 (GLP-1) receptor agonists, cardiovascular benefits tend to be greater than simple weight reduction and appear to be connected with improvements in the course of atherosclerosis [2]. Lipid-laden adipocytes secrete a myriad of chemokines that attract circulating mononuclear cells, which are responsible for low-level inflammation in adipose tissue [3]. Infiltrating monocytes become macrophages and perpetuate local inflammation, leading to the liberation of free fatty acid and insulin resistance [4]. In these circumstances, not only is the number of monocytes greater but also their phenotype is different [5]. Macrophages release an increased quantity of proinflammatory cytokines (e.g., Il-1β, TNFα) and induce substantial oxidative stress [6]. Circulating monocytes also infiltrate cholesterol-laden arterial walls, resulting in atherosclerotic plaque formation. At some point, circulating monocytes infiltrate other organs and tissues, participating, for example, in the progression of MASLD, which is a clinical entity beginning from simple liver steatosis but leading to steatohepatitis and finally liver cirrhosis [7].

GLP-1 analogs improve the course of cardiovascular and metabolic complications in obesity [8]. Additional pleiotropic effects are thought to be related to anti-inflammatory and antioxidative properties that were noted in experimental settings in HepG2 cells [9] or in adipocyte in vitro cultures [10]. But benefits of GLP-1 analogs seem to extend beyond the direct activation of GLP-1 receptors on hepatocytes or adipocytes. Attention should also be given to macrophages, which are key players in the development of atherosclerosis, low-level inflammation, and oxidative stress. As mentioned earlier, foam cells (derived from macrophages) dwell in arterial walls, while other organ-specific macrophage-derived cells (e.g., Kupffer cells) are important in the course of target organ function and damage. Macrophages are not a uniform population of cells and can possess different capabilities. Typically, classically activated macrophages (M1) are characterized by the expression of specific markers (e.g., inducible nitrite oxide synthase—iNOS), significant proinflammatory cytokine synthesis (e.g., TNFα, IL-1β), and high oxidative potential. This phenotype is connected with a response to inflammatory/bacterial stimuli (LPS, TNFα). Alternative activated macrophages (M2) are generally seen in the healing phase, have anti-inflammatory features, and are characterized by the expression of arginase 1 and mannose receptors (MRs). Previously, it was shown that an ex vivo GLP-1 agonist (exenatide) shifts human monocytes/macrophages toward an anti-inflammatory (M2) phenotype [11], which was associated with an enhancement of their antioxidant capacity [12]. However, data on the actual influence of incretin-based in vivo therapies on macrophages’ phenotype in human subjects are scarce. Such an impact might explain the link between improvements in cardiovascular and metabolic outcomes in patients treated with GLP-1 analogs. Furthermore, it might encourage further studies on pinpointing mechanisms responsible for metabolic improvements attributed to GLP-1 receptor activation in other target organs (e.g., liver).

In order to address the paucity of available data, we conceived a study to explore the effects of subcutaneously administered liraglutide (a GLP-1 analog) (up to 3.0 mg per day) in obese patients and estimated the basic features of macrophages’ phenotype, the expression of inflammatory cytokines, and the level of oxidative stress generated ex vivo by macrophages obtained prior and after 3-month liraglutide therapy.

## 2. Materials and Methods

### 2.1. Subjects

**Figure 1:**
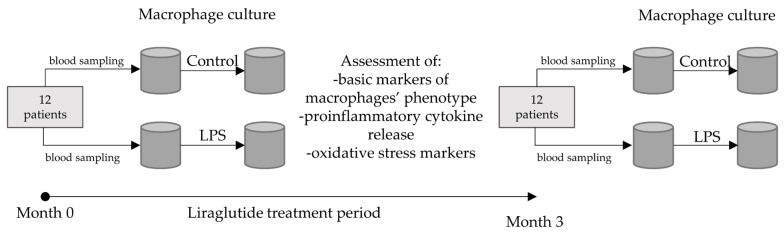
Flowchart of the study.

The study population included 12 (8 females and 4 males) obese (BMI > 30 kg/m^2^), adult, otherwise healthy subjects who were going to be treated with subcutaneous, daily liraglutide injection (up-titrated from 0.6 mg to 3 mg per day). The mean age of patients was 46.6 ± 13.6 years. The youngest participant was 26, and the oldest was 68. Patients were recruited from the Outpatient Clinic of the Department of Internal Medicine and Clinical Pharmacology (Medical University of Silesia, Katowice, Poland) and provided informed consent to participate in the study. During the course of the experiments, two blood samplings were scheduled—prior to the therapy and after three months of the treatment with liraglutide. According to Appendix A [13], Taxonomy for Study Designs, the type of experiment should be regarded as a before–after study (patient therapy) with comparative analysis of macrophages in vitro [13]. All patients completed the study period. The ethical committee of the Medical University of Silesia approved the study protocol (BNW/NWN/0052/KB1/97/23). The basic study flowchart is presented in Figure 1.

### 2.2. Procedures (Monocyte Culture, Transition to Macrophages, Challenge with LPS)

Peripheral blood monocytic cells (PBMCs) were isolated from blood that was drawn from antecubital veins via venipuncture between 8:00 and 9:00 a.m. to minimize circadian variations of leukocytes. PBMCs were separated from blood through density gradient centrifugation with Histopaque 1077 (Sigma-Aldrich, St. Louis, MO, USA), as previously described [12]. Afterwards, monocyte isolation from PBMCs was performed by the negative immunomagnetic technique with pan-B and pan-T antibodies (Invitrogen Dynal AS, Oslo, Norway). Consequently, non-stimulated mononuclear cells were acquired and placed in RPMI-1640 medium containing 10% low endotoxin fetal calf serum, 2 mM glutamine, 100 U/mL penicillin, 100 mg/mL streptomycin, and 10 ng/mL Gibco fungizone (Thermo Fischer Scientific, Inc., Grand Island, NY, USA). By means of a TC-20 automated cell counter (Bio-Rad Laboratories, Inc., Hercules, CA, USA), a constant number of 1 × 10^6^ living cells per well were placed in 12-well plastic plates (BD Biosciences, Franklin Lakes, NJ, USA) and allowed to adhere for 2 h. In the following step, the medium was exchanged with a fresh one, and cells were placed in an incubator at 37 °C in predefined conditions (a mixture of 95% air and 5% CO_2_) for 72 h with an additional medium exchange at 24 h (Heraeus, Hera-Cell, Thermo Fischer Scientific, Inc., Grand Island, NY, USA). As a result, a transformation of monocytes into macrophages occurred, which was verified under phase-contrast microscopy through visual observation of the increased cytoplasmic/nuclear area ratio of cells. Finally, the supernatant was removed, and a medium or a medium supplemented with LPS at 0.5 μg/mL concentration was added (Cat. No. 437627, Merck Sigma-Aldrich, Poznan, Poland). Experiments were performed in duplicate.

### 2.3. Analytical Methods

#### 2.3.1. RT-qPCR

The assessment of inducible nitric oxide synthase (iNOS, coded by *NOS2* gene), arginase 1 (*ARG1*), and mannose receptor (*MRC1*) was performed by means of quantitative polymerase chain reaction (qPCR). Primer sequences for *NOS2*, *ARG1*, and *MRC1* were obtained from PrimerBank database, as follows [14]: *NOS2*: forward-GCA GCC TTT GTG AAC CAA CAC; reverse-CCC CGC ACA CTA GGT AGA GA. *ARG1*: forward-GAC CCT GGG GAA CAC TAC AT; reverse-GTG CCA GTA GCT GGT GTG AA. *MRC1*: forward-TTT TTC CTT TGC CTA ATT GAA; reverse-GCT GAC ATC AGC TAC CCA TC. β-actin (*ACTB*) was used as a reference for other gene expression assessments. The PrimerBank database was also employed to acquire primer RT-QPCR sequences, as follows: *ACTB* forward-TCA TGA AGT GTG ACG TGG ACA TC; *ACTB* reverse-CAG GAG GAG CAA TGA TCT TGA TCT. Macrophage cultures on 12-well culture plates (1 × 10^6^ cells per well) were treated with 1 mL of TRI reagent (MRC Inc. Cincinnati, OH, USA) to lyse cells and obtain total RNA, which was later dissolved in 150 µL of nuclease-free water. The concentration and purity were assessed by spectrophotometer at a wavelength of 260/280 nm (BioPhotometer, Eppendorf GmbH, Hamburg, Germany). Then, 1 µg RNA was transcribed into cDNA and resuspended in a 20 µL solution. In the last stage of the procedure, a 5-fold dilution of samples was performed, as instructed by the manufacturer (GoScript Reverse Transcription System, Promega GmbH, Walldorf, Germany). RT-PCR reaction solutions consisted of 10 µL of SYBR Select Master Mix (Thermo Fisher Scientific, Warsaw, Poland), 0.2 µM of each primer (*NOS2* F/R or *ARG1* F/R or *MRC1* F/R), 2 µL of reverse transcription mixture (equivalent to 10 ng of total RNA). To estimate the level of gene expression, a Roche LightCycler 480 Instrument II (Roche Diagnostics, Warsaw, Poland) with a specific thermal profile (94 °C/3 min, then 45 cycles of 94 °C/30 s, 58 °C/30 s, 72 °C/45 s) was utilized. The products’ specificity was validated using melting curve generation. The value of the cycle threshold (CT) was assessed by measurements of increasing fluorescence in real time. Then, CT was normalized to that of *ACTB* expression and utilized for calculating relative gene expression using the 2^(–ΔΔCt)^ formula [15].

#### 2.3.2. Western Blotting

The following human-specific primary antibodies were employed: inducible nitric oxide synthase (iNOS) (Cat. No. SAB4502011, Merck Sigma-Aldrich, Poznan, Poland), arginase 1 (arg1) (Cat. No. HPA003595, Merck Sigma-Aldrich, Poznan, Poland); mannose receptor (MR) (Cat. No. AMAB90746, Merck Sigma-Aldrich, Poznan, Poland); and β-actin (ACTB) (Cat. No. SAB5600204, Merck Sigma-Aldrich, Poznan, Poland). Cells were cultured on 12-well culture plates (1 × 10^6^ cells per well) (SPL Life Sciences Co., Ltd., Pocheon, South Korea). Initially, plates were put on ice, and macrophages were rinsed with 500 µL of ice-cold PBS (phosphate-buffered saline). The protein fraction was extracted by means of 200 µL of cold RIPA (radioimmunoprecipitation assay) buffer with the addition of 1.5 µL of Halt Protease Inhibition Cocktail (1:100 *v*/*v*) per well (both chemicals from Thermo Fischer Scientific, Inc., Warsaw, Poland). The total quantity of protein was determined in each sample using the bicinchoninic acid assay (BCA assay, Merck Millipore, Poznan, Poland), and total protein concentration was estimated according to the standard curve based on a selection of solutions of bovine serum albumin (BSA) at predefined concentrations (Thermo Fisher Scientific, Inc., Warsaw, Poland). The separation of proteins from cell lysates was achieved by electrophoresis in polyacrylamide gel with the addition of ColorPlus Prestained Protein Marker (New England Biolabs, Lab-Jot, Warsaw, Poland). A total of 20 µg of total protein was placed into gel slots. Following separation, proteins were electroblotted right away onto a PVDF membrane (Merck Millipore, Poznan, Poland). Membranes were treated with a blocking solution of 3% BSA in Tris-buffered saline (1X TBS) for 2 h and subsequently placed in 3% BSA/1X TTBS (TBS with the addition of Tween-20 at final concentration 0.05%) with primary antibodies at a final dilution of 1:1000. During incubation, membranes were put on rocking platforms for 1 h at ambient temperature. After washing twice with TTBS for 10 min, an anti-rabbit IgG (whole molecule) peroxidase-conjugated, secondary antibody (Cat. No. A0545, Merck Millipore, Poznan, Poland) or anti-mouse secondary antibody (Cat. No. A4416, Merck Millipore, Poznan, Poland) was added (antibody dilution: 1:10,000 in 3% BSA/TTBS). The samples were incubated for one hour under continuous rocking. Ultimately, following three washes (twice with TTBS for 5 min. each and a single with TBS for 5 min.), a distinct chemiluminescent signal was detected (SuperSignal™ West Pico PLUS Chemiluminescent Substrate, Thermo Fisher Scientific, Inc., Warsaw, Poland). The signal coming from developed membranes was digitized by the ChemiDoc-It Imaging System (Analytik Jena, Jena, Germany). Assessments of relative optical density (ROD) corresponding to the amount of the target protein were performed using ImageJ software (version 1.53t) [16].

#### 2.3.3. Immunofluorescence Microscopic Imaging

Macrophages set for immunofluorescent imaging were seeded on black 12-well plates (Zell-Kontakt GmbH, Noerten-Hardenberg, Germany). Then, the cells were treated for 24 h with culture medium containing LPS or culture medium only as described above. To acquire an immunofluorescent image, specific primary antibodies were selected: anti-iNOS antibody (Cat. No. SAB4502012, Merck Sigma-Aldrich, Poznan, Poland) and anti-MR (Cat. No. AMAB90746, Merck Sigma-Aldrich, Poznan, Poland). At the end of the experiment, cells were thoroughly rinsed with 1 mL of PBS for 5 min at RT and immediately fixed using ice-cold methanol for at least 20 min at −20 °C or until subsequent analyses were performed. On the day of immunofluorescent labeling, cells were brought to an ambient temperature and rinsed twice with PBS for 5 min on the rocking platform. Cell permeabilization was facilitated by double incubation in a solution containing 0.1% Triton X-100 resolved in PBS (Merck, Poznan, Poland) for 5 min under continuous stirring, followed by washing the cells with PBS (5 min at RT). Non-specific binding sites were inhibited by incubation of the cells for 40 min in a blocking solution containing 3% BSA dissolved in PBS (Albumin Fraction V, Carl Roth GmbH, Germany, distributed by Linegal Chemicals, Warsaw, Poland). Concurrently, aliquots (500 µL) of selected primary antibody solutions (1:200) were prepared in the medium (2% BSA, 0.1% Triton X-100 in PBS). Subsequently, cells were left for incubation with antibodies for 1 h at room temperature. Then, primary antibody solutions were discarded, and culture plates were washed twice with 0.1% Triton X-100 in PBS. Afterwards, equal amounts (500 µL) of fluorescently labeled secondary antibody solution (1:300) were added into the wells for 1 h at RT and protected against daylight, as follows: Anti-Rabbit IgG (H+L), CF™488A F(ab′)2 fragment of antibody produced in goat (No. SAB4600234, Merck, Sigma-Aldrich, Poznan, Poland) or Anti-Mouse IgG (H+L), F(ab′)2 fragment, CF™647 antibody produced in goat (Cat. No. SAB4600351, Merck, Sigma-Aldrich, Poznan, Poland). Finally, wells were rinsed twice with PBS/0.1% Triton X-100 and once with PBS. The fluorescent images were analyzed and viewed under a Delta Optical IB-100 microscope equipped with an epifluorescence module (Delta Optical, Nowe Osiny, Poland).

#### 2.3.4. ELISAs

ELISA kits were acquired from a local distributor (Merck Sigma-Aldrich, Poznan, Poland) and employed for measuring the concentration of tumor necrosis factor α (TNFα) and interleukin-1β (IL-1β) (Cat. No. RAB0476 and RAB0273) in culture media obtained from experiments. The reading of optical density was done on a microplate reader (xMark™ Microplate Absorbance Spectrophotometer, Bio-Rad, Hercules, CA, USA) set at a 450 nm wavelength. The experiments were performed in duplicate. Sensitivity values for the tests were as follows: IL-1β (>0.3 pg/mL) and TNFα (>30 pg/mL). Intra-assay CVs for both cytokines were <10%.

#### 2.3.5. ROS and Malondialdehyde Assays

ROS levels were analyzed by means of a commercially attainable kit (Fluorometric Intracellular ROS Kit, Cat. No. MAK143, Merck Sigma-Aldrich, Poznan, Poland). The assay detects predominantly superoxide and hydroxyl radicals. Cells (5 × 10^4^ cells per well) were seeded on a 96-well plate in 90 µL of culture medium. Then, 100 µL of a Master Reaction Mix was added to each well and incubated for 1 h. Then, oxidative stress was induced by the addition of 10 µL of hydrogen peroxide (0.3%). The incubation lasted for 30 min. The fluorescence intensity was measured on an xMark™ Microplate Absorbance Spectrophotometer (BioRad Laboratories, Hercules, CA, USA) at 520 nm. Results are expressed as Relative Units (RU) derived from observed optical density. Assays were performed in duplicate.

In the evaluation of lipid peroxidation, a thiobarbituric acid (TBA) method was used (Lipid Peroxidation (MDA) Assay Kit, Cat. No. MAK085, Merck Sigma-Aldrich, Poznan, Poland). Cells were cultured on 12-well plates and lysed with 300 µL of lysis buffer, which then was transferred to vials for centrifugation at 13,000 g for 10 min. Then, 200 µL aliquots were mixed with 600 µL of TBA solution and incubated at 95 °C for 1 h. Once the samples reached ambient temperature, 200 µL of each reaction mixture was transferred to a 96-well plate for analysis. The intensity of fluorescence was evaluated using an xMark Microplate Absorbance Spectrophotometer (Bio-Rad Laboratories, Hercules, CA, USA) at 532 nm. Results are expressed in Relative Units (RU) stemming from the measured optical density. Assays were done in duplicate.

### 2.4. Statistical Analysis

The sample size was estimated according to previous in vitro data (exenatide was able to reduce TNFα by around 30% expression in cultured macrophages [11]), which showed that the sample necessary to provide statistically meaningful results should not be less than 11 (with type I (α) error < 0.05 and statistical power set at 80%). The normality of the distributions was analyzed by means of Shapiro–Wilk’s test. Data are expressed as means ± SEM. Comparisons between groups were performed using one-way ANOVA, followed by Dunn’s tests or Kruskal–Wallis, followed by multiple Mann–Whitney with Bonferroni adjustments according to variable distribution. A *p*-value < 0.05 was considered statistically significant. Data analysis was performed using Statistica 13.0 (StatSoft, Cracow, Poland).

## 3. Results

### 3.1. Basic Phenotypical Features of Macrophages During the Course of In Vivo Treatment with Liraglutide and In Vitro Challenge with LPS

At the beginning of the experiments, we assessed the impact of liraglutide therapy on basic markers of macrophage phenotype. Those experiments included both the comparison of the expression of markers of classical as well as alternative activation prior to and after the 3-month introduction of liraglutide. We also evaluated whether the 3-month incretin-based therapy affected the response to a proinflammatory stimulus with LPS.

#### 3.1.1. Marker of Classical Activation (M1): Inducible Nitric Oxide

**Figure 2:**
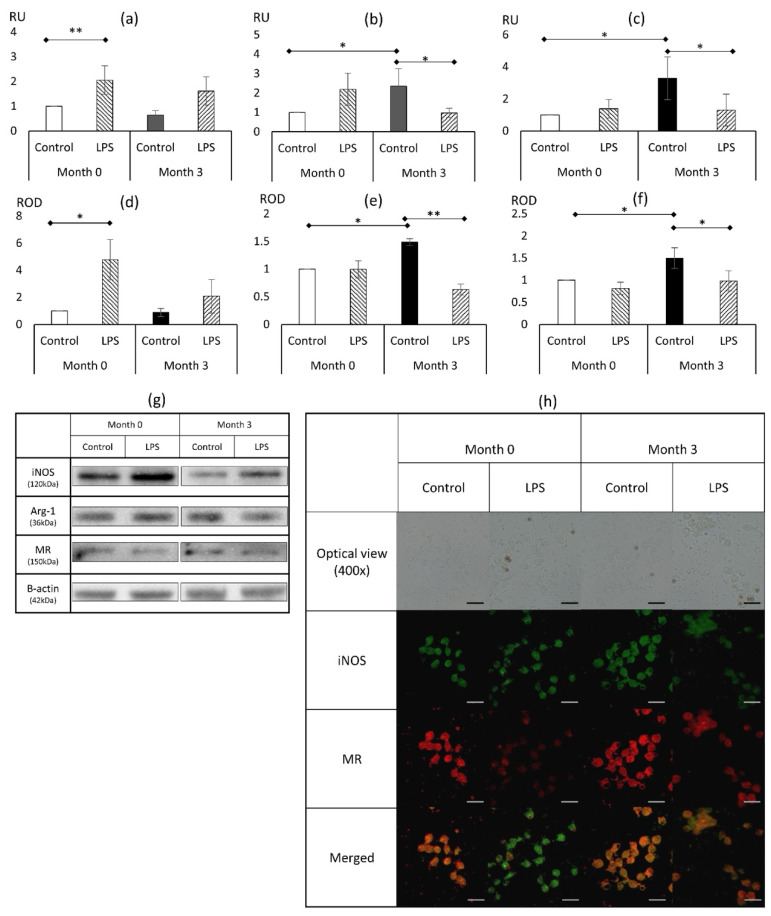
Basic phenotypical features of macrophages during the course of in vivo treatment with liraglutide and ex vivo challenge with LPS. The expression of mRNA for *NOS2* (**a**), *ARG1* (**b**), and *MRC1* (**c**). Protein expression of iNOS (**d**), arg1 (**e**), and MR (**f**). Representative Western blots for assessment of protein expression (**g**). Immunofluorescent staining of macrophages for iNOS and MR (**h**). Bar represents 50 µm (*n* = 3–7). *—*p* < 0.05; **—*p* < 0.01. Abbreviations: *ARG1*/arg1—arginase 1; *NOS2*/iNOS—inducible nitric oxide; LPS—lipopolysaccharide; *MRC1*/MR—mannose receptor; ROS—relative optical density; RU—relative units.

In experiments involving the assessment of the reactivity of macrophages prior to the liraglutide therapy, a significant two-fold (*p* < 0.01) elevation in *NOS2* mRNA and a nearly 5-fold (*p* < 0.05) increase in protein expression during LPS stimulation was noted (Figure 2a,d). There have been no statistically significant differences in the expression of mRNA and protein between samples taken prior to and at the end of therapy. However, the impact of the liraglutide therapy was noticeable and depicted by the loss of the inducibility of iNOS at mRNA and protein levels in cells derived from patients during the 3-month liraglutide treatment.

#### 3.1.2. Markers of Alternative Activation (M2): Arginase 1, Mannose Receptor

Treatment with liraglutide resulted in a significant elevation of expression of both assessed markers of alternative activation, i.e., arg1 (Figure 2b,e) and MR (Figure 2c,f). *ARG1* mRNA level was doubled (*p* < 0.05), and the protein level increased by 49% (*p* < 0.05). The protein level for MR was elevated to a similar extent (by nearly 50%; *p* < 0.05), whereas mRNA expression was tripled (*p* < 0.05). Further experiments showed that strong inflammatory stimulus (LPS) can abolish the impact of liraglutide therapy, leading to a significant drop in the expression of both arg-1 and MR to levels comparable to those seen in control cultures prior to liraglutide therapy.

### 3.2. Markers of Proinflammatory Response

#### 3.2.1. TNFα

**Figure 3:**
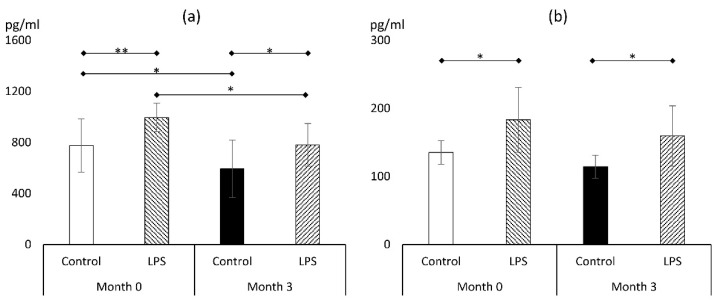
Markers of proinflammatory response: TNFα (**a**) and IL-1β (**b**) (*n* = 7). *—*p* < 0.05; **—*p* < 0.01. Abbreviation: LPS—lipopolysaccharide.

LPS was a solid stimulant of TNFα release, both prior to and after the liraglutide treatment (Figure 3a). The 3-month therapy with liraglutide resulted in a reduction in TNFα release to culture medium in macrophages that were not subjected to LPS (593 ± 226 vs. 776 ± 209 pg/mL; *p* < 0.05). This phenomenon was also observed in macrophages exposed to LPS for 24 h, resulting in a reduction in TNFα concentration (995 ± 112 vs. 781 ± 167 pg/mL; *p* < 0.05). Therefore, regardless of LPS use, liraglutide therapy resulted in a reduction in TNFα levels of 21–23%. As a result, the release of TNFα during LPS exposure at the end of the therapeutic period was reduced to levels similar to those seen in unstimulated macrophages prior to the treatment (781 ± 167 vs. 776 ± 209 pg/mL; *p* > 0.05).

#### 3.2.2. IL-1β

LPS significantly increased the amount of IL-1β in the culture medium in macrophages derived before and after the 3-month liraglutide treatment (Figure 3b). Contrary to TNFα findings, the therapy with liraglutide failed to affect IL-1β release both in unstimulated (135 ± 17 vs. 114 ± 17 pg/mL; *p* > 0.05) and LPS-exposed macrophages (183 ± 47 vs. 159 ± 43 pg/mL; *p* > 0.05). Nevertheless, at the end of the treatment period, the IL-1β release to culture medium in macrophages exposed to LPS was similar to unstimulated macrophages prior to liraglutide therapy, which may suggest preventive action against inflammatory stimuli.

### 3.3. Markers of Oxidative Stress

#### 3.3.1. Reactive Oxygen Species (ROS)

**Figure 4:**
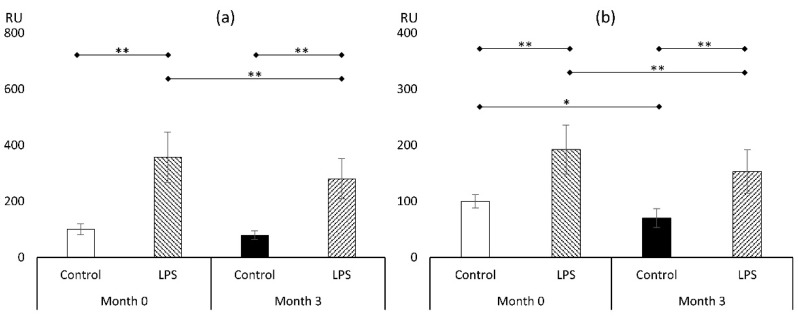
Markers of oxidative stress: reactive oxygen species (**a**) and malondialdehyde (**b**) (*n* = 8). *—*p* < 0.05; **—*p* < 0.01. Abbreviation: LPS—lipopolysaccharide.

As shown in Figure 4a, macrophages harvested from subjects prior to the initiation of liraglutide and treated with LPS showed significant elevation in ROS level, reaching 357 ± 88% (*p* < 0.01) compared to control cultures devoid of LPS. Similar effects were noted after the liraglutide therapy, resulting in an increase in ROS level after LPS (79 ± 15% vs. 280 ± 72%; *p* < 0.01). After the course of incretin-mimicking drug for three months, there has been no impact on ROS level in cultures not treated with LPS, but a statistically significant reduction in ROS level in cells that have been exposed to LPS (280 ± 72% vs. 357 ± 88%; *p* < 0.01) was noted.

#### 3.3.2. Malondialdehyde (MDA)

Another marker of oxidative stress that we employed in the experimental setting was MDA (Figure 4b). It corresponds with prolonged oxidative stress influence on lipids, resulting in lipid peroxidation. Here, we showed that LPS treatment at the beginning of the study elevated the MDA level nearly two-fold (192 ± 44%; *p* < 0.01). Similar to the ROS findings, the MDA level was reduced in LPS-treated macrophages (153 ± 39% vs. 192 ± 44%; *p* < 0.01). But contrary to experiments estimating the ROS level, a significant reduction in the level of MDA in cells that have not been exposed to LPS was also noted (70 ± 17%; *p* < 0.05). Those findings reflect that the antioxidative properties of the 3-month course of treatment with liraglutide in cultured macrophages reduced the acute oxidative stress response (as depicted by ROS experiments), which diminished oxidative stress legacy, as shown by experiments on MDA accumulation in cells not treated with LPS.

## 4. Discussion

Incretin-based therapies lead to effective weight reduction, which is accompanied by several benefits, including cardiovascular. Cardiovascular complications are generally the result of atherosclerosis. Macrophages participate in nearly every pathophysiological step of atherosclerotic plaque formation. However, they are not a uniform population of cells and may show different features when exposed to various conditions, both in vitro and in vivo [17,18]. Human macrophages express GLP-1 receptors on their surface, providing a target for incretin-based drugs [19,20]. The major intracellular mechanism connected with the GLP-1 receptor agonism seems to be connected with increased cAMP availability and the activation of protein kinase A [21], which, in turn, inactivates NFκB [11], resulting in a plethora of effects [22]. NFκB is essential in the development of inflammation (including the expression of several cytokines) and oxidative stress due to the induction of NADPH oxidase—a major source of ROS. Here, we showed that a 3-month treatment of obese patients with liraglutide can change macrophage phenotype mainly by skewing the population toward features of the less inflammatory population (M2), with an accompanying reduction in TNFα secretion and reduced oxidative stress.

In previous studies, the in vitro exposure of human monocytes/macrophages to exenatide (another GLP-1 receptor agonist) showed a significant drop in the level of TNFα secreted to culture media [11], while liraglutide led to an effective reduction in the expression of TNFα and IL-1 in cultured rat chondrocytes [23]. In vivo animal studies also seem to support the impact of liraglutide on the release of proinflammatory cytokines. The multifactorial effects of liraglutide also encompassed a reduction in TNFα level, which might be considered an important factor in the protective effects of the drug in the course of organ damage in diabetes mellitus [24]. However, the results were based on the liver expression of TNFα. Anti-inflammatory properties of liraglutide, described by a reduction in serum IL-1β level, were also attributed to improved insulin sensitivity in diabetic mice [25]. Other studies, including ischemia/reperfusion in C57BL/6J mice, showed effective attenuation of TNFα and IL-1β after pretreatment of animals with liraglutide. Furthermore, 3-month therapy with liraglutide in patients with DM2 significantly reduced TNFα concentration (26.3 ± 5.0 vs. 19.7 ± 3.6 pg/mL; *p* = 0.012), and this effect was thought to be independent of improved glycemic control [26]. Data from studies assessing the impact of in vivo liraglutide therapy on human macrophage cytokine release are lacking. However, our data not only confirm the anti-inflammatory potential of liraglutide but also show that it directly affects macrophages during in vivo treatment. Furthermore, in our study (selective measurement of cytokine release by macrophages), we were able, to some extent, to exclude potential bias resulting from weight loss during the liraglutide treatment, which is connected with a reduced inflammatory state in adipose tissue. Elevated TNFα levels seem to be connected with the progression of atherosclerosis. Therefore, tapering the macrophage TNFα release may be responsible for the improvements seen in the course of several diseases (e.g., atherosclerosis, steatohepatitis, insulin resistance).

The results of the current study show that the oxidative stress level in cultured macrophages was diminished. Therapy with liraglutide resulted in the reduction of MDA levels without the addition of LPS to culture media, whereas ROS generation was diminished by liraglutide only in macrophages exposed to LPS. As mentioned earlier, incretin-based therapies, via the inhibition of NFκB, reduce the expression of NADPH oxidase, resulting in a reduction in the oxidative burden. Additionally, an increase in antioxidant enzyme expression was noted in experiments involving exendin-4 (a GLP-1 receptor agonist) [27]. Findings from ROS experiments might represent a reduction in the acute response of macrophages, whereas MDA reduction might show the chronic impact of liraglutide on low-level oxidative stress. Others have reported that the combined treatment with liraglutide and metformin of diabetic patients affected the level of oxidative stress represented by a reduction in the MDA level (by approx. 15%) and an elevation in the SOD activity [28]. However, in this setting, due to the use of metformin, the net effect of incretin-based therapy was difficult to measure. This issue was addressed in a 12-month study by Lambadiari et al. [29]. During the follow-up of patients with diabetes, a gradual reduction in the extent of oxidative stress markers was noted. At the end of the study, the MDA level was significantly reduced by 30%. Contrary to our experimental setting, both of these studies relied on plasma sampling. The elevated level of oxidative stress markers might be a potential link with the progression of atherosclerosis. In mice studies, it was shown that liraglutide improves the course of aortic aneurysms if administered at the early phases of its formation due to a significant reduction in MDA level and mononuclear cell infiltration [30]. This study substantiates the benefits of the early administration of GLP-1 analogs that might prevent atherosclerotic plaque formation and progression. Therefore, in clinical practice, early initiation of therapy should be advocated prior to the development of complications (i.e., preferably in obese patients before the development of diabetes, hypertension, etc.).

Our experimental setting showed the flexibility of mononuclear cells. At the end of the experiment, macrophages expressed greater amounts of markers of alternative activation. Until now, phenotype-modifying properties of incretin-based therapies were reported in in vitro and ex vivo macrophage cultures [31]. Chronic inflammation and the classical activation of macrophages is typical for obesity [32]. Those phenomena are responsible for the progression of atherosclerosis and its complications. It seems promising that the therapy that is focused on GLP-1 receptor agonists may influence macrophages toward a protective phenotype, which might be one of the reasons for cardiovascular risk reduction during therapy with GLP-1 analogs in obese subjects [33]. This phenotypic shift seems to result from the activation of the protein kinase A pathway and its downstream protein kinase B/Akt [11], as well as the upregulation of signal transducer and activator of transcription 3 (STAT3) [20].

Limitations of the study should be kept in mind. The procedure of harvesting and culturing monocytes and their transformation to macrophages is by itself stressful for cells and might result in unwanted cell activation, which must be taken into consideration. Interpatient variability was also clearly visible in our study and should also be taken into account during the interpretation of data as well as a relatively small sample size. Furthermore, the potential influence of therapeutic lifestyle changes that were also recommended to all participants of the study cannot be completely excluded. Nevertheless, the strength of the study lies in its in vivo nature of therapy with liraglutide in the clinical setting of obesity and the direct assessment of the reactivity of macrophages in vitro.

## 5. Conclusions

In summary, we report that the 3-month therapy of obese patients with liraglutide affects macrophage phenotype and possesses anti-inflammatory (reduced TNFα expression) and antioxidative (reduced MDA) properties.

## Data Availability

Data are available on reasonable request from corresponding authors.
